# Anterior Total Hip Arthroplasty With Bulk Femoral Head Autograft in a Patient With Camurati-Engelmann Disease

**DOI:** 10.1016/j.artd.2021.03.011

**Published:** 2021-04-14

**Authors:** Adam J. Taylor, Robert P. Runner, Donald B. Longjohn, Soheil Najibi

**Affiliations:** a Department of Orthopaedic Surgery, Rancho Los Amigos National Rehabilitation Center, Downey, CA, USA; b Department of Orthopaedic Surgery, Keck Medical School of University of Southern California, Los Angeles, CA, USA

**Keywords:** Camurati-Engelmann disease, Total hip arthroplasty, Hip dysplasia, Femoral head autograft, Direct anterior approach

## Abstract

Camurati-Engelmann disease (CED) is an extremely rare, sclerosing bone disorder of intramedullary ossification with only 300 reported cases worldwide. The pathogenesis is related to activating mutations in transforming growth factor beta 1, which results in bilateral, symmetric hyperostosis affecting primarily the diaphysis of long bones. Despite effective pharmacological treatment options, the diagnosis of CED is problematic owning to its rarity and variability of clinical presentation. We present a patient with known CED with advanced early hip osteoarthritis, secondary to underlying hip dysplasia, for which she underwent a successful total hip arthroplasty via a direct anterior approach with the use of bulk femoral head autograft to reconstruct her native acetabulum.

## Introduction

Camurati-Engelmann disease (CED), also known as progressive diaphyseal dysplasia, is a rare, autosomal dominant, disorder characterized by osteosclerosis of the long bones and the skull. Patients with CED can present with a range of clinical manifestations; however, symptom onset is typically within the first 30 years of life with limb pain, muscle weakness, and a waddling gait as dominating features [1]. Characteristic radiographic findings include hyperostosis of the diaphysis of the long bones, which may progress to the metaphysis and, rarely, to the epiphyses [1,2].

The pathogenesis of CED is associated with abnormalities in intramembranous ossification caused by activating mutations in transforming growth factor beta 1 (TGFB1) [3]. There are only 300 reported cases of CED worldwide, which makes the identification of CED problematic despite characteristic radioclinical findings [1,2]. With availability of effective treatment modalities, such as corticosteroids and angiotensin II type 1 receptor antagonists, early diagnosis is important to improve clinical outcomes of affected patients [4].

Arthroplasty in patients with CED is typically for those with advanced degenerative changes, and given the rarity of the disorder, only 2 case reports of total hip arthroplasty (THA) have been previously presented [5,6]. We report on a patient with CED with advanced early hip osteoarthritis, secondary to underlying hip dysplasia, for which she underwent a successful THA via a direct anterior approach (DAA) with the use of bulk femoral head autograft (FHA) to reconstruct her dysplastic acetabulum and bring her hip center down to an anatomic inferomedial position.

The patient was informed that data concerning the case would be submitted for publication, and she provided consent.

## Case history

A 48-year-old female with known CED presented with chronic left hip pain, which progressively worsened over the previous year. She was diagnosed with CED at the age of 26 years after experiencing several years of pain in her extremities and easy fatiguability, which eventually prompted her to present to the emergency department. Radiographs of her lower extremities were performed, and given the atypical findings, she was referred to her primary care physician for further workup. Subsequent genetic testing was performed and confirmed her diagnosis of CED, and treatment with oral prednisone was initiated.

Upon presentation to the orthopedic clinic, she lived a semi-sedentary lifestyle and was only able to ambulate 2 to 3 blocks in the community with support of a front wheeled walker. She described her symptoms as severe left groin pain, rated 8/10, that was worse with activities, especially and when rising from the seated position. She had been taking 10 mg of oral prednisone and diclofenac daily for several years before presentation, with minimal improvement in her symptoms. She denied any history of trauma, surgery, or corticosteroid injections to her left hip.

The patient’s body mass index at initial presentation was 23.1 kg/m^2^. On examination, she had severe pain with range of motion to her hip with hip flexion to 80°, internal rotation to 10°, external rotation to 20°, abduction to 25°, and adduction to 30°. Her gait examination was significant for antalgic gait with a positive Trendelenburg sign. She exhibited 4/5 weakness to her hip abductors and was otherwise neurovascularly intact distally. Her preoperative clinical limb length discrepancy (LLD) was 2 cm. The patient’s preoperative hip dysfunction and osteoarthritis outcome score for joint replacement (HOOS, JR) raw score was 20 with an interval score of 25.103.

**Figure 1:**
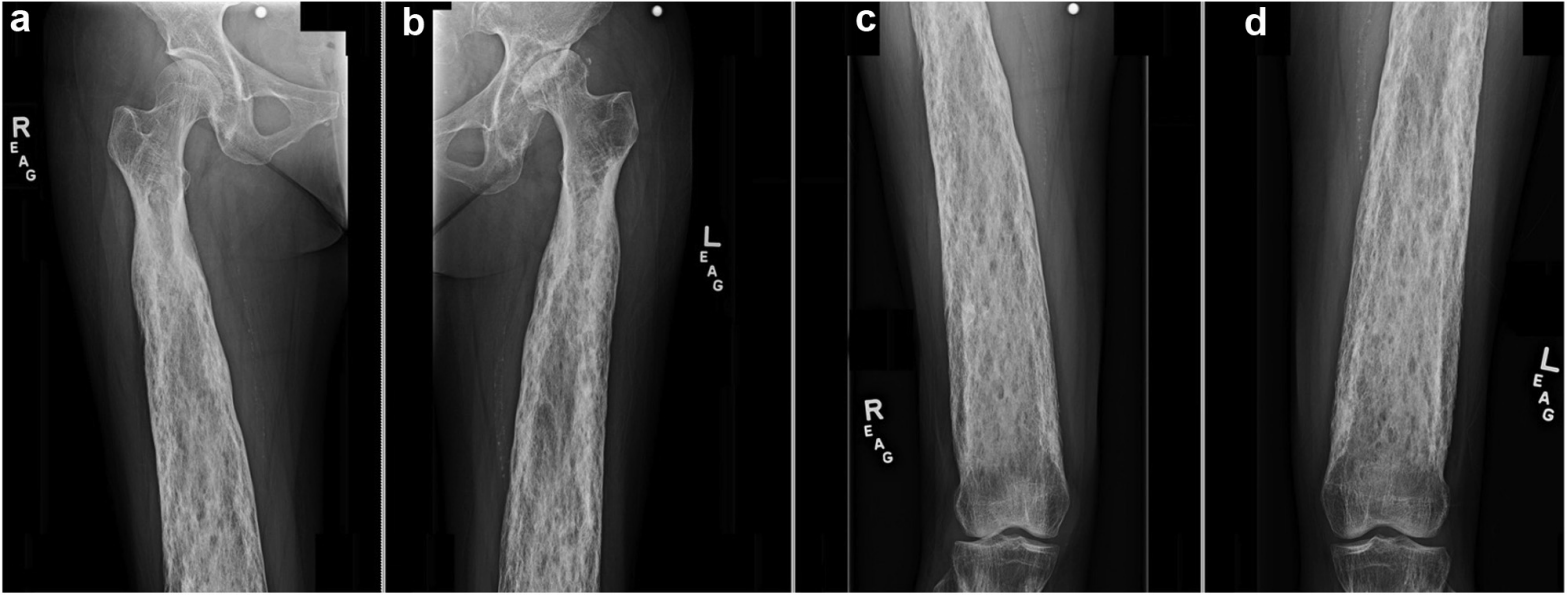
Preoperative anteroposterior radiographs of the right (a, c) and left (b, d) femurs demonstrating symmetric, bilateral cortical thickening and broadening of the diaphysis.

**Figure 2:**
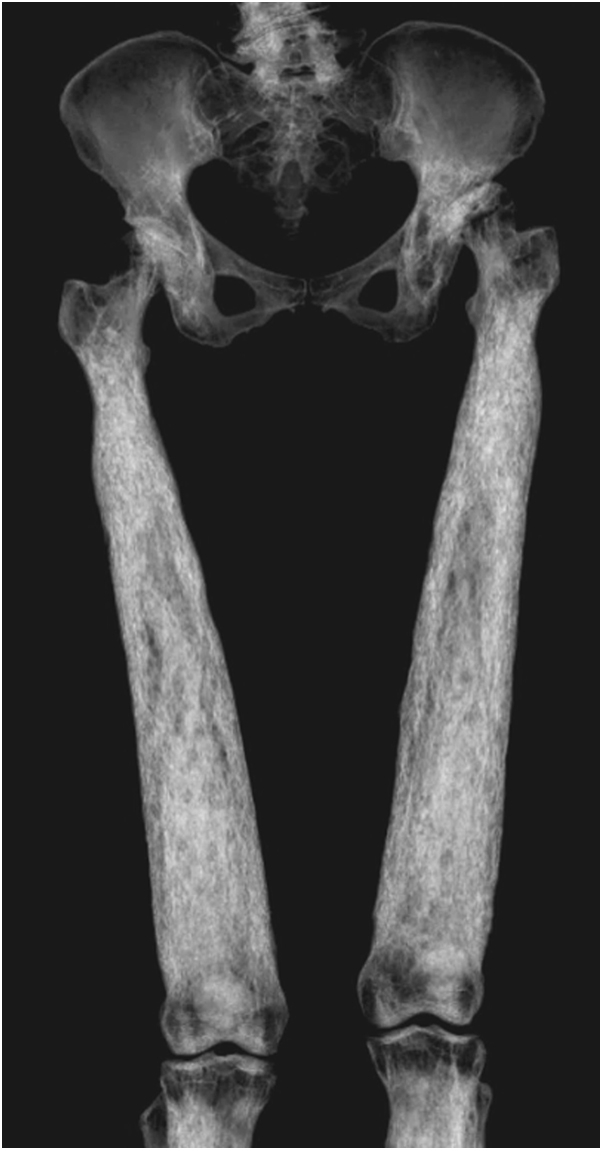
Three-dimensional reconstruction of computerized tomography (CT) scan demonstrating diffuse hyperostosis, with broadening of the femoral diaphysis.

**Figure 3:**
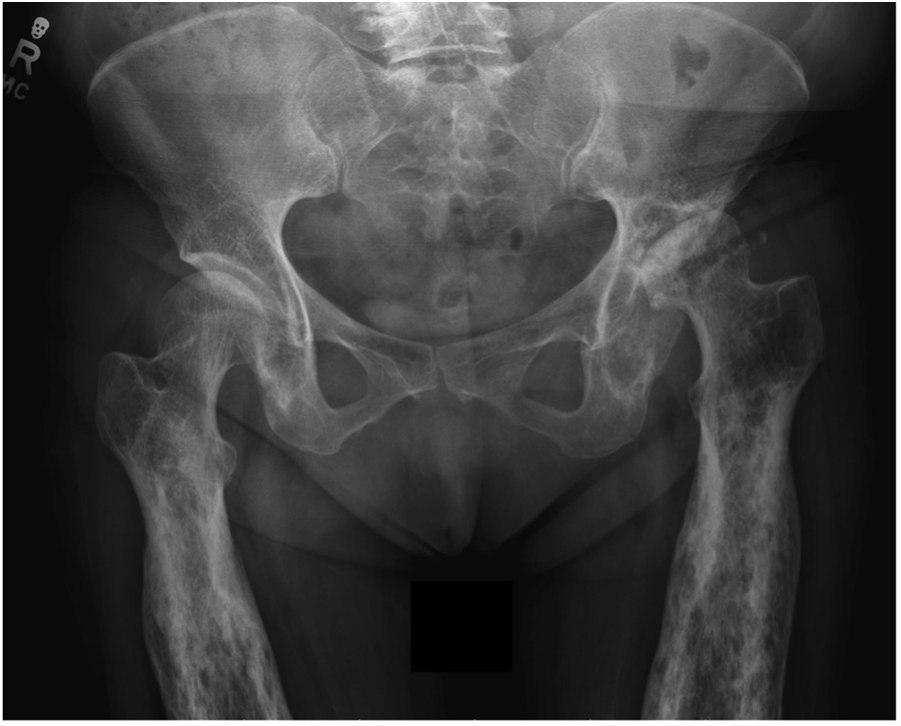
Preoperative anteroposterior pelvis radiograph showing advanced degenerative changes to the left hip with a large subchondral cyst in the roof of the acetabulum and a Paprosky IIB defect in the lateral rim of the acetabulum. Proximal and lateral migration of the left hip is also noted, consistent with Crowe II dysplasia.

**Figure 4:**
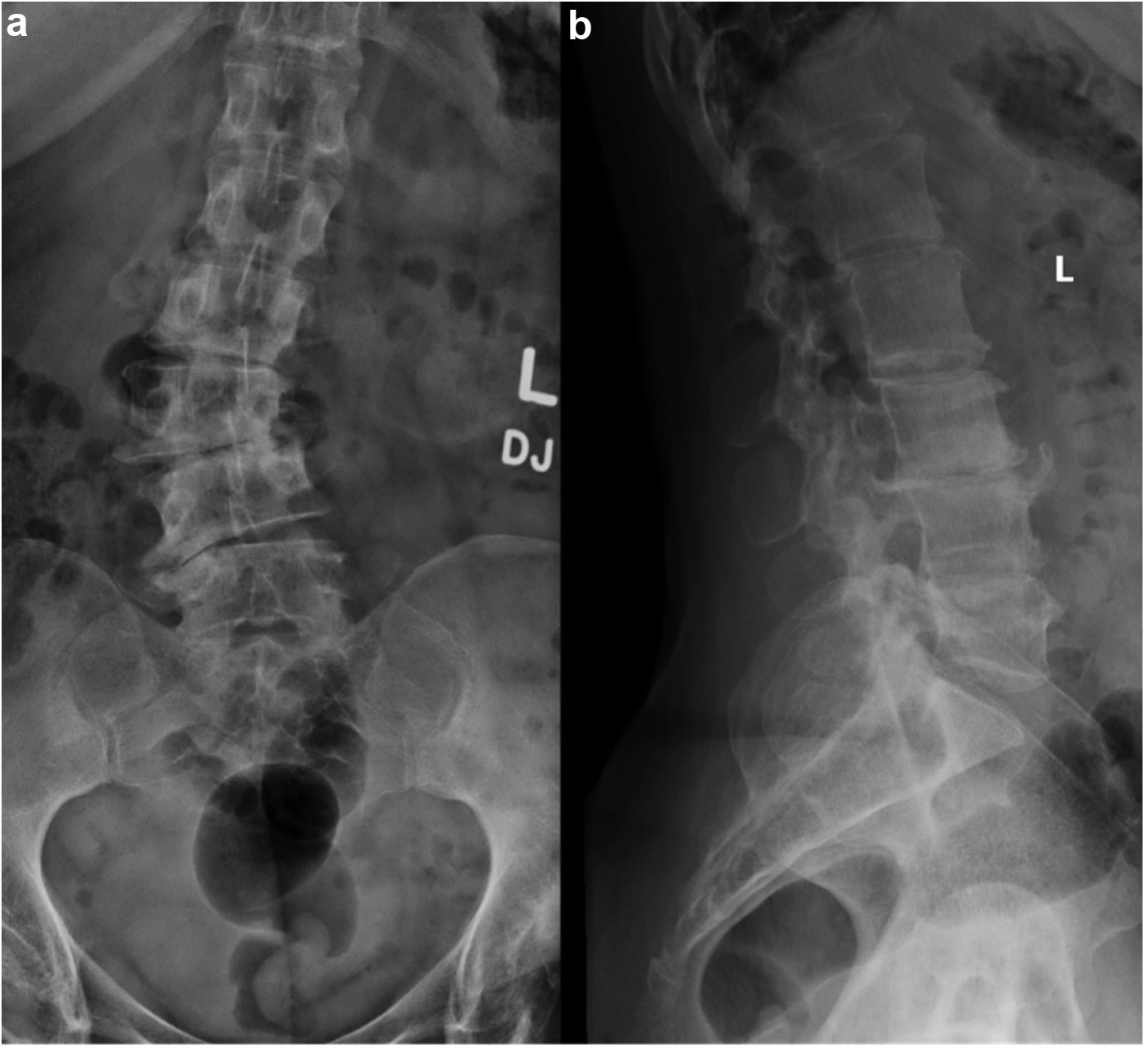
Preoperative anteroposterior (a) and lateral (b) radiographs of the lumbar spine demonstrating degenerative lumbar scoliosis.

Preoperative radiographs demonstrated hyperostosis of her bilateral femurs with broadening of her diaphysis and narrowing of the medullary canal, consistent with CED (Figure 1, Figure 2). Radiographs of the patient’s left hip demonstrated significant osteoarthritic changes with a large subchondral cyst noted in the roof of the acetabulum and a Paprosky IIB defect in the lateral rim of the acetabulum (Fig. 3). Proximal and lateral migration of the left hip with a break in Shenton’s Arc was also noted on radiographs, consistent with Crowe II hip dysplasia [7]. In addition, the patient was noted to have degenerative lumbar scoliosis without coronal or sagittal plane imbalance (Fig. 4). She was evaluated by the surgical spine service, and given the asymptomatic nature of the patient’s lumbar spine disease, and that if future surgery was to be done for her lumbar spine it would not have a major change in her sagittal or coronal balance, it was decided to proceed with left THA.

### Preoperative planning

The patient was evaluated by her primary care physician and anesthesiologist to ensure medical optimization before surgery. As she had been taking oral corticosteroids daily for several years and had attempted to wean to as low a dose as possible, it was planned for her to continue these medications through her surgery date, with stress doses on the day of surgery.

**Figure 5:**
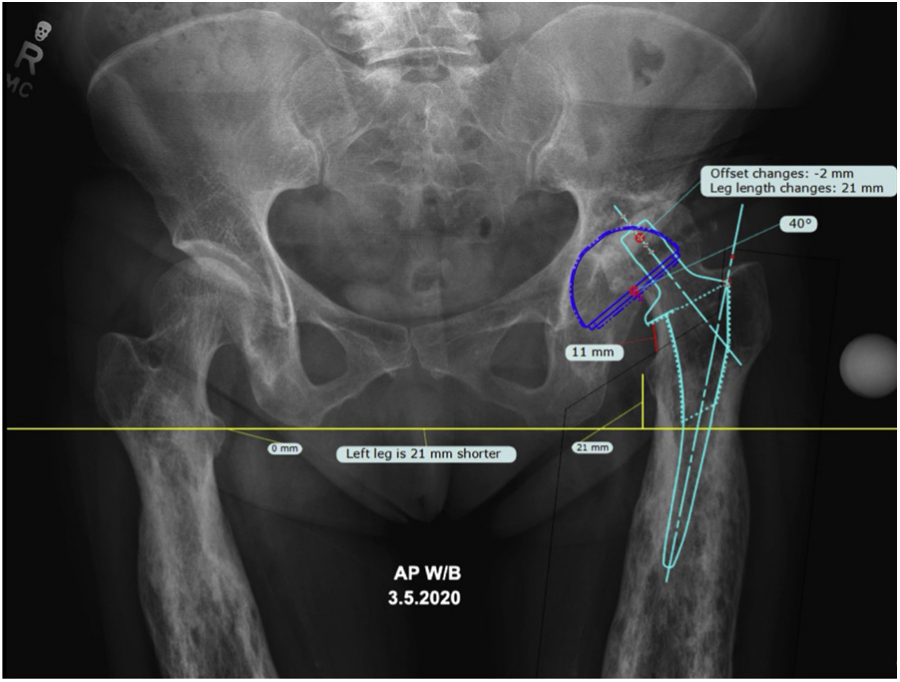
Preoperative anteroposterior pelvis radiograph with templating using the TraumaCad-software system (Voyant Health) demonstrating a 21-mm leg length discrepancy.

The senior surgeon routinely uses a DAA for all THA cases with uncemented titanium porous-coated acetabular components (G7 OsseoTi; Zimmer-Biomet, Warsaw, IN). Preoperative radiographs were templated via the TraumaCad-software system (Voyant Health, Petah Tikva, Israel) (Fig. 5). Her LLD measured -21 mm on her left side preoperatively. Given the dysplastic nature of the acetabulum with significant lateral uncoverage, a universal small fragment system with long pelvic screw set (Depuy Synthes, Raynham, MA) was made available for possible bulk FHA. Porous tantalum and titanium acetabular augments were available as well.

**Figure 6:**
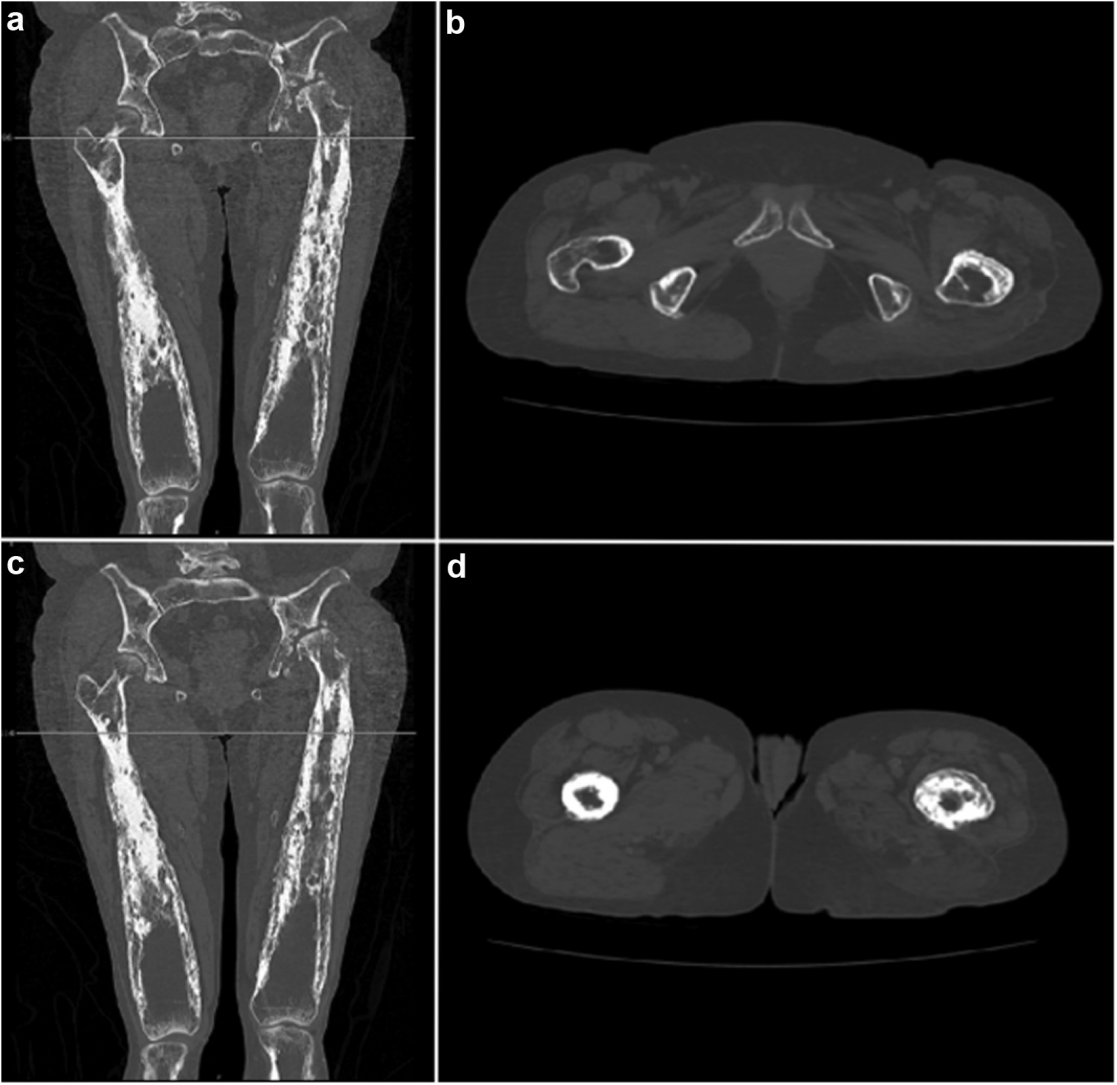
Preoperative computerized tomography (CT) scans in the coronal (a, c) and axial (b, d) planes demonstrating diffuse cortical thickening and narrowing of the medullary canal.

A computerized tomography scan was done preoperatively to assess for the intramedullary canal size and metaphyseal-diaphyseal mismatch (Fig. 6). Significant narrowing of her intramedullary canal with relative sparing of her metaphysis was noted, as is typical of CED, and planned for a primary metaphyseal fitting stem. To ensure proper stem fit and axial and rotational stability, the triple-tapered, collared stem with proximal hydroxyapatite coating (ACTIS; DePuy Synthes, Raynham, MA) was templated as the first option because of its reduced distal tip with options for flexible distal reamers if diaphyseal expansion was required. However, multiple stem systems were made available depending on intraoperative findings. A femoral cable system and cemented components were also made available on the day of surgery, given the unknown bone quality preoperatively.

### Operative technique

The patient was positioned supine on a fracture table (Hana Orthopedic Surgery Table; Mizuho OSI, Union City, CA). The standard DAA (Hueter interval) was used. During exposure, significant scarring of tissues was noted with adherence of the tensor fasciae latae to the surrounding fascia. The ascending branch of the lateral femoral circumflex artery was identified and coagulated, the capsule exposed, and arthrotomy performed. Before femoral neck osteotomy, the hip was dislocated without difficulty, the ligamentum teres was cut, and all soft tissue attachments of the femoral head were released. The hip was then reduced, and the neck osteotomy was performed based on the preoperative template relative to the saddle at the neck and great trochanter and distance proximal to the lesser trochanter. After femoral neck osteotomy, the head was carefully removed with bone forceps, rather than a corkscrew, to ensure preservation of the structural integrity of the femoral head in case autografting was required. The acetabulum was visualized and found to have large uncontained superolateral defect measuring 12 × 20 mm and a large subchondral cyst in the acetabular roof. Given these findings, a bulk FHA was planned to reconstruct the inferomedial native hip center and provide additional lateral coverage of the cup.

**Figure 7:**
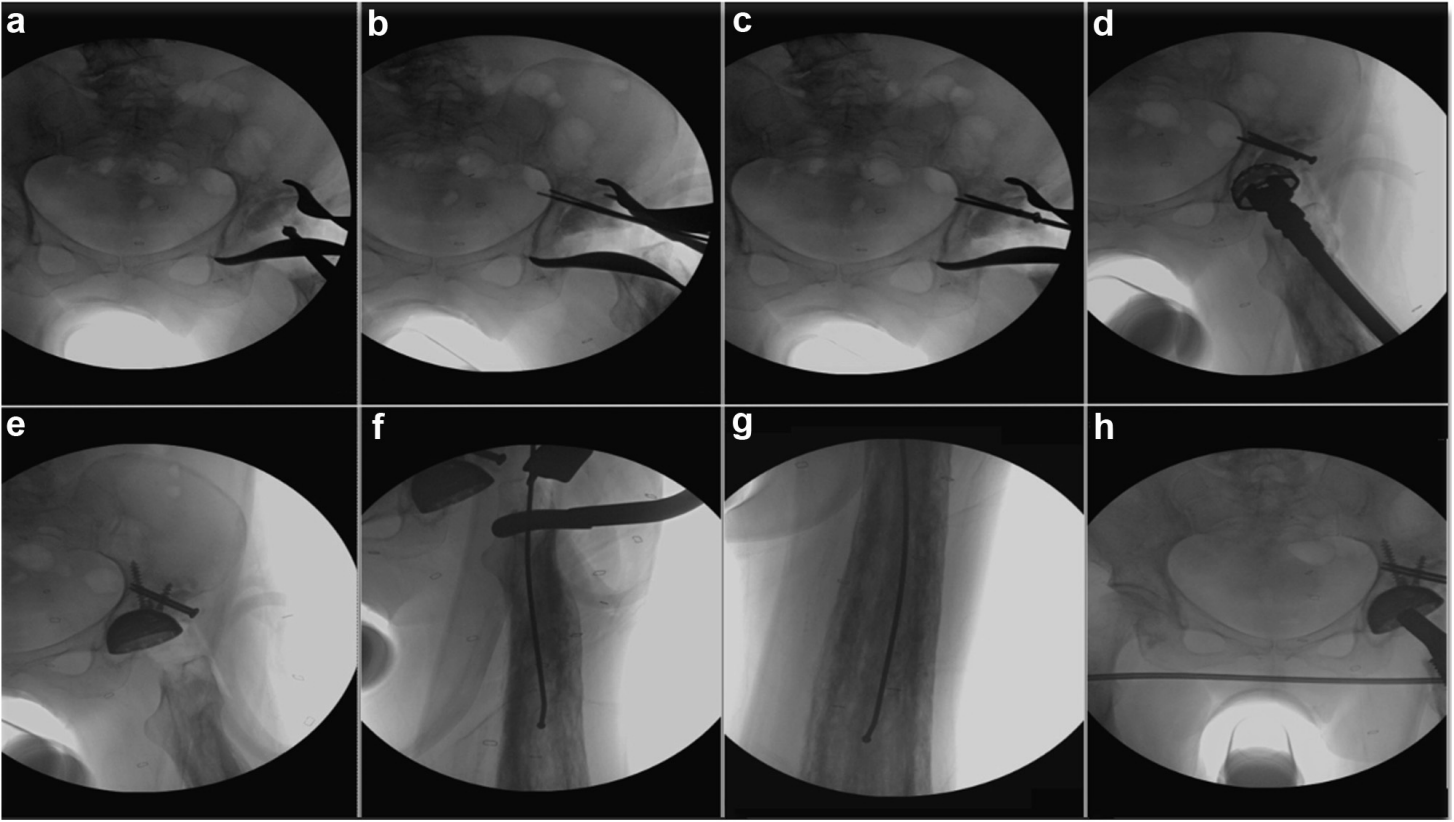
Intraoperative fluoroscopy depicting surgical technique. (a) Hemispherical graft is held in place with a ball spikes; (b) graft is provisionally fixed with 2 2.7-mm long drill bits; (c) drill bits exchanged with 3.5-mm fully threaded screws penetrating the outer and inner cortex of the ilium; (d) Sequential reaming of the acetabulum is performed until appropriate rim fit into the autograft and host bone is achieved; (e) three 6.5-mm acetabular screws are placed through the cup, 2 of which were able to capture both graft and host bone; (f-g) guide wire for flexible reamer passed confirming an intramedullary possible via orthogonal images; (h) trial reduction showing restoration of leg lengths and offset.

FHA preparation and fixation was achieved by the following steps: 1) The native superolateral defect of the acetabulum was reamed, to prepare a vascular bed of bone for graft incorporation; 2) the femoral head was decorticated with an oscillating bone saw and shaped with a burr into a hemispherical shape to match the convexity of the now reamed pseudoacetabulum; 3) bulk FHA was oriented in an inlay manner such that the subchondral portion of the graft was in maximum contact with the acetabular defect; 4) the graft was held in place with a ball spike and then provisionally fixed with 2 2.7-mm drill bits; 5) positioning and stability of the graft was confirmed via fluoroscopy, and drill bits were exchanged sequentially for 3.5-mm cortical screws; 6) sequential reaming in an ideal inferomedial position for the acetabulum was performed and underreamed by 1 mm for appropriate rim fit into the autograft and host bone; 7) a press-fit acetabular shell (46 mm) was inserted under fluoroscopic guidance at approximately 40° of abduction and 25° of anteversion with the pelvis oriented on fluoroscopy to match her preoperative standing anteroposterior pelvis radiograph; 8) three 6.5-mm fully threaded cancellous acetabular screws (15 mm, 25 mm, and 40 mm) were placed through the shell into the posterosuperior quadrant, 2 of which captured both graft and host bone for additional graft stability (Fig. 7). Mechanical stability of the cup was confirmed, and a neutral, vitamin E infused, highly cross-linked polyethylene liner (Vivacit-E; Zimmer-Biomet, Warsaw, IN) was then inserted (32 mm inner diameter).

The femoral exposure and instrumentation were achieved by performing routine releases of the superior capsule to expose and elevate the proximal femur. The canal was initially opened with a rongeur and rasp; however, the medullary canal and proximal femur were extremely sclerotic and would not accommodate the starter broach. The proximal femur was instead opened with a long high-speed burr to remove areas of sclerotic bone that impeded entry of the femoral broach. A long ball tipped guide wire was then passed and confirmed to be within the medullary canal on orthogonal images (Fig. 7). Flexible reamers were then used to enlarge the medullary canal distally, starting with an 8.5-mm reamer and then proceeding in 1-size increments up to a size of 12 mm. The femoral canal was then broached in 1-size increments until axial and rotational stability was achieved (size 5 broach). The standard neck and a trial femoral head (32 mm + 5) were placed, and trial reduction was performed with the hip noted to be stable to physiologic and supraphysiologic motion. Using contralateral hip fluoroscopic overlay images, restoration of her leg length and offset was achieved using this construct and corrected her preoperative LLD (Fig. 7). The final components, including a ceramic femoral head (BIOLOX delta; Depuy Synthes, Raynham, MA), were then inserted, and the wound was irrigated and closed in the routine manner.

### Postoperative course

**Figure 8:**
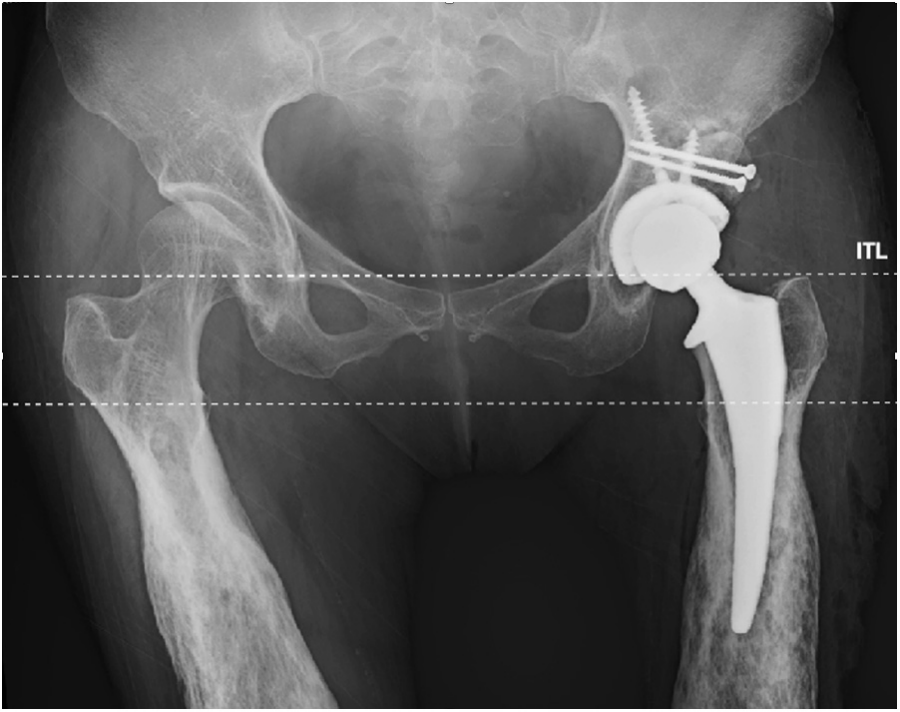
Postoperative anteroposterior radiograph demonstrating restoration of leg lengths and offset. ITL, interteardrop line.

Postoperative radiographs demonstrated proper placement of the femoral and acetabular implants with near symmetric restoration of her LLD (Fig. 8). The patient was made partial weight-bearing (30 lb total weight) on the ipsilateral extremity for 6 weeks, to allow incorporation of the autograft. She was started on enteric coated aspirin (325 mg, PO BID) on postoperative day 1 for deep vein thrombosis prophylaxis. Her hospital course was uneventful and did not require transfusion, and she was discharged home on postoperative day 2. She followed up at routine intervals of 2 weeks, 6 weeks, 3 months, and 1 year postoperatively without significant adverse events. She was able to wean off her walker at 8 weeks postoperatively and was ambulating without assist devices by 4 months postoperatively. At her most recent follow-up, she had no notable pain in her left hip, ambulated without a limp, and was able to perform all activities of daily living without limitations. She is extremely satisfied with the results, with a most recent HOOS, JR raw score of 1 and an interval score of 92.340.

## Discussion

CED is an extremely rare, sclerosing bone disorder of intramedullary ossification with an estimated incidence of 1 in 1,000,000 worldwide [8]. The pathogenesis is related to increased TGFB1 activity, with most cases (82.2%) associated with missense mutations in the latency associated peptide of exon 4, resulting in premature activation of the mature peptide.

At normal physiological levels, TGFB1 acts as a coupling factor resulting in an overall response of increased bone secretion and decreased bone resorption [3]. Activating mutations in TGFB1, therefore, causes bilateral, symmetric hyperostosis which primarily affects the long bones (94%), the pelvis (63%), and the skull (54%) [1,2].

Most patients with CED are symptomatic (74%), with most symptoms related to hyperostosis [3]. Limb pain is the most common symptom (68%), which is often observed in patients during their first decade of life [1,9]. Skull-based manifestations, such as hearing loss (19%), headache (10%), and exophthalmos (8%), are less common and typically occur at a later onset [9]. TGFB1 is also a known inhibitor of myogenesis and adipogenesis; therefore, patients may also present with a waddling gait (48%), easy fatigability (44%), muscle weakness (39%), reduced muscle mass (39%), and reduced subcutaneous fat (21%) [1,10,11].

Considering its progressive nature, early identification and treatment of CED may improve patient outcomes. Today, diagnosis is typically dependent on clinical and radiographic findings and confirmed via genetic testing, although scintigraphy can assist in early detection before changes are seen on radiographs [2,12]. At the time of diagnosis, patients are often started on oral corticosteroids, which have been shown to improve clinical symptoms without regression of radiographic manifestations [1,13,14]. Losartan, an angiotensin II receptor antagonist, has also been shown to decrease limb pain and increase exercise capacity in patients with CED through the downregulation of TGFB1 receptors, although further research is needed to validate its use [15,16]. Surgery is typically reserved as a last resort in the treatment of CED, wherein procedures such as reaming the medullary canal have been reported to have temporary effects on pain relief [17,18].

Given the rarity of the disease, only 2 case reports of THA in patients with CED have been reported [5,6]. To our knowledge, however, this is the first reported case report of THA in a patient with CED that was carried out via a DAA, and the first to be performed with the use of bulk FHA. In this case, the patient demonstrated advanced osteoarthritis of her left hip, secondary to underlying hip dysplasia, which was refractory to nonoperative measures. A THA with reconstruction of the native acetabulum was recommended. Extensive preoperative planning, including computerized tomography scan and radiographic templating, was critical to assess bone quality, canal dimensions, and ensure restoration of mediolateral offset and LLD. A DAA was used as it is the preferred approach of the senior surgeon and because it would facilitate the use of intraoperative fluoroscopy, which provided real time feedback on guide wire placement and reaming the medullary canal.

Similar to the study by Ge et al. [5], we found a proximal metaphyseal fitting stem to be most appropriate, given the narrowing of the medullary canal and relative sparing of the metaphysis, that is, typical of CED. Surgeons should also anticipate proximal sclerosis in the metaphysis, however, and be prepared to remove these sclerotic regions to allow entry of a broach; in this case, we found long high-speed burrs to be useful. In addition, flexible reamers were necessary in this case to open the medullary canal and allow for distal fit of the stem. Unlike Ge et al., we opted not to place a prophylactic cable around the femur given the excellent bone quality found intraoperatively, although this approach may be considered based on surgeon preference and intraoperative findings.

### Current controversies and future considerations

In terms of the patient’s hip dysplasia and associated acetabular bone deficiencies, we find bulk FHA to be useful in re-establishing the anatomic hip center and providing additional bone stock. Although the use of FHA has been historically controversial because of long-term concerns of graft resorption and component loosening [19], many recent studies have shown high long-term survival rates using cementless hemispherical porous-coated sockets [[20], [21], [22], [23]]. Alterative techniques, such as a creation of a high hip center, have also demonstrated success in these cases [[24], [25], [26]]; however, other studies have shown diminished hip biomechanics [27] and increased rates of aseptic loosening [28,29] when the cup is placed outside the native acetabulum. Furthermore, we advocate for the use of a DAA in these cases as it provides direct exposure of the acetabulum and facilitates the use of intraoperative fluoroscopy, which has been associated with decreased cup malpositioning [30] and accurate leg length restoration [31,32]. Further studies are necessary to assess the outcomes of FHA in THA via the DAA.

## Summary

This case report describes a 48-year-old female with CED who underwent a successful THA via a DAA with the use of bulk FHA to reconstruct her native acetabulum. CED is an extremely rare, autosomal dominant, sclerosing bone disorder of intramembranous ossification. Limb pain and radiographic findings of symmetric hyperostosis with cortical thickening of long bones are characteristic features. Corticosteroids or losartan are the current pharmacological treatment options, but limitations still exist regarding their safety and efficacy. Surgery is considered the last resort in treatment for CED, with only 2 reported cases of THA in the literature. When approaching these cases, metaphyseal fitting stems are recommended with a low threshold to ream distally and avoid potting a stem, given the typical metaphyseal-diaphyseal mismatch. In cases with associated hip dysplasia, bulk FHA can be considered a reliable and cost-effective option for reconstructing dysplastic acetabula. Future studies are necessary to evaluate the outcomes of bulk FHA in THA using a DAA.

## Key points

1. Consider CED on your differential when a patient presents with limb pain and radiologic findings of symmetric hyperostosis of long bones with broadening of the diaphysis and narrowing of the medullary canal.
2. When approaching THA in patients with CED, metaphyseal fitting stems are recommended with a low threshold to ream distally and avoid potting a stem, given the typical metaphyseal-diaphyseal mismatch.
3. Although the diaphysis is typically the most sclerotic region in patients with CED [1,2], surgeons should anticipate metaphyseal sclerosis and be prepared to remove these sclerotic regions to allow entry of a broach; in this case, we found long high-speed burs to be useful.
4. In patients with underlying hip dysplasia, the use of bulk FHA is a well-described technique that can not only reconstruct the native hip center but also restore acetabular bone stock. Key technical factors for FHA include 1) initial graft and cup stability with screw placement through both the graft and the cup; 2) graft orientation in an inlay manner; 3) press-fitting the cup to provide an impacted cancellous surface; 4) the use of porous-coated cups to facilitate bone ingrowth [20].

## Conflicts of interest

The authors declare that they have no known competing financial interests or personal relationships that could have appeared to influence the work reported in this article. Funds were received for publication from Rancho Research Institute.

## Appendix A Supplementary data

Conflict of Interest Statement for Taylor

Conflict of Interest Statement for Longjohn

Conflict of Interest Statement for Runner

Conflict of Interest Statement for Najibi
