# Supplementary material for: Anterior Total Hip Arthroplasty With Bulk Femoral Head Autograft in a Patient With Camurati-Engelmann Disease
Source: Arthroplast Today. 2021 Apr 14;8:204–10. doi: 10.1016/j.artd.2021.03.011 (PMC8076616; doi:10.1016/j.artd.2021.03.011)
Supplement: Conflict of Interest Statement for Taylor [file mmc1.pdf]

CONFLICT OF INTEREST STATEMENT

American Association of Hip and Knee Surgeons

(Adopted from the American Academy of Orthopaedic Surgeons disclosure statement)

The following form **must be filled out completely and submitted by each author (example, 6 authors, 6 forms). All items require a response. If there is no relevant disclosure for a given item, enter "*None.*"**

Manuscript Title

1. Royalties from a company or supplier (The following conflicts were disclosed)

   None.

2. Speakers bureau/paid presentations for a company or supplier (The following conflicts were disclosed)

   None.

3A. Paid employee for a company or supplier (The following conflicts were disclosed)

   None.

3B. Paid consultant for a company or supplier (The following conflicts were disclosed)

   None.

3C. Unpaid consultants for a company or supplier (The following conflicts were disclosed)

   None.

4. Stock or stock options in a company or supplier (The following conflicts were disclosed)

   None.

5. Research support from a company or supplier as a Principal Investigator (The following conflicts were disclosed)

   None.

6. Other financial or material support from a company or supplier (The following conflicts were disclosed)

   None.

7. Royalties, financial or material support from publishers (The following conflicts were disclosed)

   None.

8. Medical/Orthopaedic publications editorial/governing board (The following conflicts were disclosed)

   None.

9. Board member/committee appointments for a society (The following conflicts were disclosed)

   None.

Each author must sign AND print or type his/her name, date and submit a separate form

In addition, one BLINDED Conflict of Interest form (no author names used) should be submitted per manuscript with all author disclosures.

Adam Taylor	DocuSigned by: Adam Taylor 4B206ACD2C7049A...	1/23/2021
Author Name (Print or Type)	Author Signature	Date
